# Phylogenetic groups, extended-spectrum β-lactamases and metallo-β-lactamase in *Escherichia coli *isolated from fecal samples of patients with diarrhea in Iran 

**Published:** 2015

**Authors:** Hesam Alizade, Fatemeh Fallah, Reza Ghanbarpour, Mohammad Reza Aflatoonian, Hossein Goudarzi, Hamid Sharifi

**Affiliations:** 1 *Department of Microbiology, International Branch, Shahid Beheshti Universityof Medical science, Tehran, Iran*; 2 *Department of Microbiology, Faculty of Medicine, Shahid Beheshti University of Medical Science, Tehran, Iran*; 3 *Molecular Microbiology Department, Faculty of Veterinary Medicine, Shahid Bahonar University, Kerman, Iran*; 4 *Research Center for Tropical and Infectious Diseases, Kerman University of Medical Sciences, Kerman, Iran*; 5 *Department of Food Hygiene and Public Health, Faculty of Veterinary Medicine, Shahid Bahonar University of Kerman, Iran*; 6 *Research Center for Modeling in Health, Institute for Futures Studies in Health, Kerman University of Medic al Sciences, Iran*

**Keywords:** *Escherichia coli*, Diarrhea, Extended-spectrum β-lactamase, Metallo-β-lactamases

## Abstract

**Aim:**

The aims of this study were to investigate the phenotypic and genotypic of extended-spectrum β-lactamase (ESBL) and metallo-β-lactamases (MBLs) and determine phylogenetic background *E. coli* isolates from fecal samples of patients with diarrhea in Kerman, southeast of Iran

**Background:**

The emergence of ESBLs and MBLs-producing *E. coli *caused problems in antibiotic treatments. *E. coli *strains can be assigned to four main phylog-groups, including: A, B1, B2 and D.

**Patients and methods:**

*E. coli* isolates (n=216) were obtained from fecal samples of patients with diarrhea between June and December 2013. ESBLs and MBLs were confirmed by disk-diffusion and broth micro-dilution methods. Using PCR, the ESBL-positive isolates were screened to determine the phylo-groups and the presence of *bla*_CTX-M-15_, *bla*_OXA-1_, *bla*_PER-1_, *bla*_VIM_ and *bla*_IMP_ genes.

**Results:**

ESBL-positive isolates (n= 56) were detected. Among ESBL-positive isolates, 51 isolates were positive for *bla*_CTX-M15_ and one isolate was positive for both *bla*_CTX-M-15_ and *bla*_OXA-1 _genes. None of the isolates were positive for *bla*_PER-1_, *bla*_VIM_ and *bla*_IMP_ genes. PCR assay for phylotyping of isolates indicated that the isolates were belonged to groups A (54.16%), B1 (11.11%), B2 (12.96%) and D (21.75%). The isolates possessed *bla*_CTX-M-15_ gene were belonged to A (35 isolates), B1 (5), B2 (3) and D (8) phylo-groups.

**Conclusion:**

Our results indicate that *bla*_CTX-M-15_ gene is widespread among diarrheagenic *E. coli* isolates. ESBL-producing *E*. *coli *isolates were disseminated among a diversity of phylo-groups. Further studies are necessary to identify the ESBL genes in relation to phylogenetic groups.

## Introduction

 Diarrheal diseases are one of the important causes of morbidity and mortality in developing countries. *Escherichia coli* (*E. coli*) is identified as a significant cause of sporadic and outbreaks of diarrhea worldwide (1, 2). The emergence and distribution of multi-drug resistant strains of *E. coli* is complicating the treatment of various serious infections (3). 

In recent years, the emergence of extended-spectrum-β-lactamase (ESBL)-producing *E. coli* has been one of the most significant epidemiologic changes in infectious disease (4). 

Types of enzymes conferring resistance to β-lactam antibiotics have emerged due to antibiotic selection pressure; most alarming are the ESBLs produced by enteric pathogens that have spread throughout the world (5).

ESBLs confer resistance to antibiotics including, third and fourth-generation cephalosporins and monobactams. CTX-M enzyme types have emerged worldwide among *Enterobacteriaceae *species, in particular *E. coli*. The CTX-M-1 β-lactamase is the most prevalent classes of enzymes throughout the world with the CTX-M-15 being the most recognized variants. CTX-M-14 and CTX-M-15 clusters are widely diffused among humans in Europe (3). During the last four years descriptions of new OXA-type β-lactamases have increased considerably. Thus, Poirel et al. reported in 2010 that 147 OXA enzymes had been assigned to this group. Today this number has reached 227 OXA-type beta-lactamases (6). OXA-type β-lactamases have been detected in many Gram-negative bacteria, in particular *Pseudomonas aeruginosa. *However, among OXA-type β-lactamase, OXA-1 has been found in 1-10% of *E. coli *isolates (7). PER-1 is widely distributed in Turkey and share 86% amino acid homology with PER-2, which is found mostly in South America, several countries in Europe and the Far East (8).

Metallo-β-lactamases (MBLs) are founded increasingly in Gram-negative organisms and are recognized mostly in *Pseudomonas *species. MBLs hydrolyze all β-lactams, including carbapenems. Among *Enterobacteriaceae* species, the *bla*_IMP_ and *bla*_VIM_ genes have been identified throughout the world (9).

The *E*. *coli *species are genetically diverse, and strains have been divided into four major phylogenetic groups, the most frequent ones being A, B1, B2, and D. The most diarrheagenic *E. coli *strains belong to group D and the commensal strains to groups A and B1. However, ESBL-producing *E*. *coli *is genetically various (10).

 The purposes of the current study were to determine the phylogenetic groups and assess phenotypic and genotypic of ESBL and MBL-producing *E*. *coli *isolates from fecal samples of patients with diarrhea in Kerman, southeast of Iran.

## Patients and Methods

**Bacterial isolates**

The research study was approved by the department of Microbiology, International Branch, Shahid Beheshti University of Medical science, Tehran, Iran. *E. coli* isolates (n=216) were obtained from patients with diarrhea who referred to different clinical laboratories and hospitals in Kerman city (southeastern of Iran) between June to December 2013. Diarrheic samples were identified as the incidence of watery and unformed stools with or without one of the following symptoms: fever, nausea, abdominal cramps, and tenesmus. Their age ranged from <5 years old (69), 5 to 15 years old (44), 15 to 40 years old (64) and 40 to 80 years old (39). Stool specimens were directly streaked onto Mac-Conkey agar (Biolife Laboratories, Milan, Italy) for isolation of *E. coli*. After overnight incubation at 37°C, lactose-fermenting colonies were selected and identified by the biochemical and bacteriological tests.

**PCR**

Whole-cell DNA was extracted using the lysis method. ESBL-positive isolates were screened by PCR for the carbapenemase-encoding genes, *bla*_IMP_ and *bla*_VIM _(11). Detection of other β-lactamase genes such as *bla*_CTX-M-15_, *bla*_OXA-1_ and *bla*_PER-1_ was performed with amplification conditions, as described previously (12-14). All *E. coli* isolates were assigned to phylogenetic groups using the multiplex PCR method (15). On the basis of presence or absence of the *chuA*, *yjaA *genes and an anonymous DNA fragment, TspE4.C2 by multiplex PCR, the *E. coli* isolates were assigned to one of four groups (A, B1, B2 and D). The specific primers used for detecting sequences encoding MBLs, ESBLs and phylogenetic groups are presented in Table 1.

**Modified Hodge test**

Phenotypic confirmatory test for carbapenemase production in *E. coli* isolates was done by modified Hodge test using meropenem (10 ug) as the indicator disc according to CLSI guidelines 2013 (16).

**Antimicrobial susceptibility testing**

 The determine antimicrobial susceptibilities of the studied isolates than ceftazidime (30 mg), ceftazidime–clavulanic acid (30/10 mg), cefotaxime (30 mg), cefotaxime–clavulanic acid (30/10 mg), cefepime (30 mg), C (10 mg) and aztreonam (30 mg) were done by using the disk diffusion method as defined by the Clinical and Laboratory Standards Institute (CLSI 2013) Quality controls were conducted using the reference strains, including: *E. coli* ATCC 25922, *E. coli *ATCC 35218 and *P. aerug*inosa ATCC 27853.

**Broth micro-dilution method**

The MICs of the following drugs were determined for isolates resistance against at least one of the antimicrobial drug by disc-diffusion method; cefotaxime (1 μg/mL), cefotaxime-clavulanic acid (0.25/4–64/4 μg/mL), ceftazidime (1 μg/mL), ceftazidime-clavulanic acid (0.25/4–128/4 μg/mL), cefepime (1 μg/mL), imipenem (1 μg/mL) and meropenem (1 μg/mL). The results of this study were interpreted, using the criteria of the Clinical and Laboratory Standards Institute (CLSI 2013) for broth micro-dilution (16). 

## Results

From the 216 *E. coli* isolates, 56 (25.92%) ESBL-positive isolates were identified by confirmatory tests (double-disc synergy test (DDST) as a standard disc-diffusion assay and broth micro-dilution) according to CLSI 2013. 

**Table 2:** Distribution of diarrhea and ESBL-positive isolates in relation to phylogenetic groups

	A no (%)	B1 no (%)	B2 no (%)	D no (%)	Total no (%)
Diarrhea isolates	117 (54.16)	24 (11.11)	28 (12.97)	47 (21.76)	216 (100.00)
ESBL-positive isolates	38 (67.86)	4 (7.14)	6 (10.71)	8 (14.29)	56 (100.00)

**Table 3:** Distribution of β-lactamase genes in relation to phylogenetic groups

	A no (%)	B1 no (%)	B2 no (%)	D no (%)	Total no (%)
*bla*_CTX-M-15_	35 (68.62)	5 (9.80)	3 (5.89)	8 (15.69)	51 (100.00)
*bla*_CTX-M-15_, *bla*_OXA-1_	1 (100.00)	-	-	-	1 (100.00)
Total	36 (69.23)	5 (9.61)	3 (5.76)	8 (15.38)	52 (100.00)

**Figure 1:**
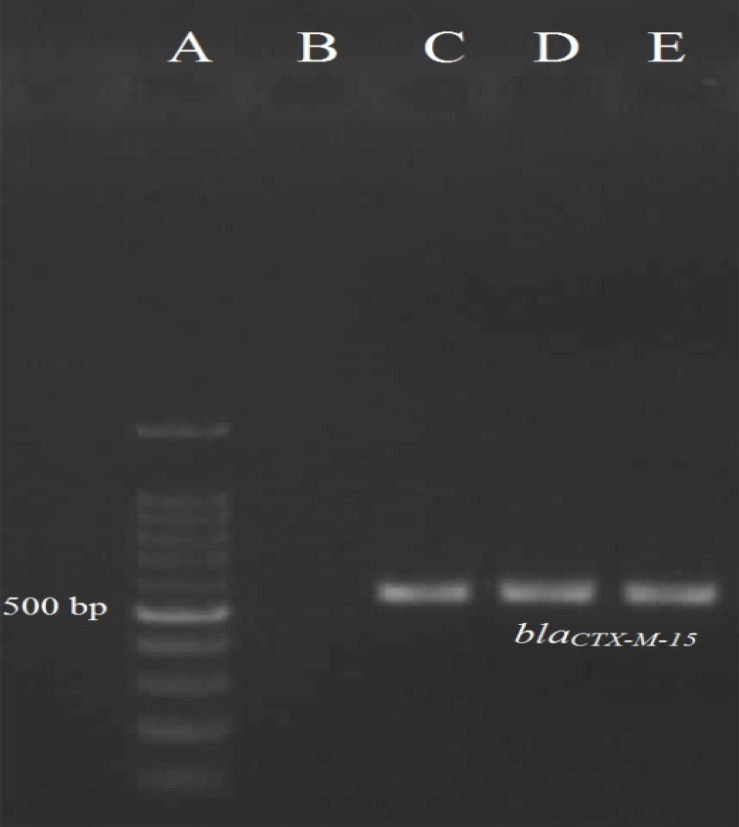
The PCR result for *bla*_CTX-M-15_ gene. A: the marker 100 bp; B: negative control *E. coli *ATCC 25922; C: positive control *Klebsiella pneumoniae *ATCC 700603; D, E: the positive isolates for *bla*_CTX-M-15_ gene

**Figure 2:**
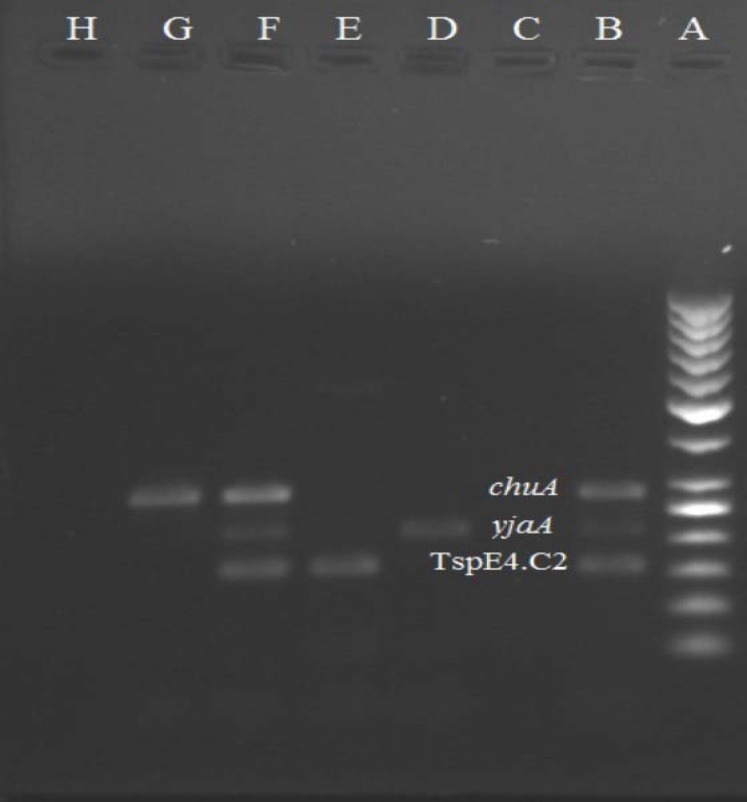
The PCR result for *bla*_OXA-1 _gene. A: the marker 100 bp; B: positive control *Klebsiella pneumoniae *ATCC 700603; C, D: the positive isolates for *bla*_OXA-1 _gene; E: negative control *E. coli *ATCC 25922

Among ESBL-positive isolates, 51 isolates (91.07%) were positive for *bla*_CTX-M-15_ and one isolate (1.78%) was positive for both *bla*_CTX-M-15_ and *bla*_OXA-1 _genes (Figure 1 and 2). The survey of ESBL-positive isolates indicated that none of the isolates were positive for *bla*_PER-1_, *bla*_VIM_ and *bla*_IMP_ genes.

**Figure 3:**
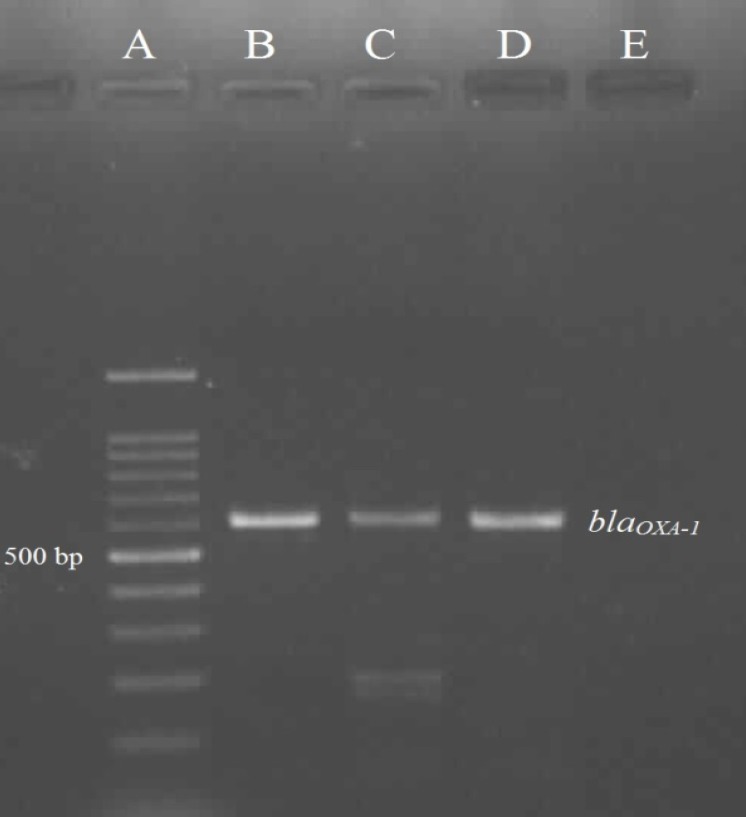
Multiplex PCR results for the detection of diarrhea *E. coli *isolates phylogenetic groups. A: ladder 50 bp; B: positive control *E. coli *ECOR62; C: negative control *E. coli *MG1655; D: A phylo-group; E: B1 phylo-group; F: B2 phylo-group; G: D phylo-group.

PCR assays for phylotyping of all isolates indicated that the isolates were belonged to phylo-groups: 117 (54.16%) isolates in A, 24 (11.11%) in B1, 28 (12.96%) isolates in B2 and 47 (21.75%) isolates in D (Figure 3). There were more phylogenetic group A and fewer group B1 isolates from ESBL-positive isolates than from non-ESBL-positive isolates (Table 2). The β-lactamase genes positive isolates belonged to four phylogroups. Fifty one positive isolates for *bla*_CTX-M-15_ gene belonged to A (35 isolates), B1 (5), B2 (3) and D (8) phylo-groups. One *bla*_CTX-M-15_ and *bla*_OXA-1 _positive isolate belong to A phylo-group (Table 3).

 All *E. coli* isolates were screened for carbapenemase production using the modified Hodge test. None of the isolates product this enzyme.

 According to the results of disk diffusion method, from 216 studied isolates 88 (40.74%) isolates were resistance to cefotaxime. Different percentages of antibiotic resistance were recorded against ceftazidime 55 isolates (25.46%), aztreonam 48 isolates (22.22%), cefepime 30 isolates (13.88%) and imipenem 6 isolates (2.77%).

The minimum inhibitory concentration of 88 *E. coli* isolates resistance against at least one of the antimicrobial drug by disc-diffusion method was done to five antibiotics. Base on the guidelines of CLSI 2013, the isolates of *E. coli *tested were resistant to cefotaxime (61 isolates), ceftazidime (38), cefepime (29) and imipenem (2) All strains were sensitive to meropenem. 

## Discussion

Extended-spectrum-β-lactamase and metallo-β-lactamases are reported increasingly in Gram-negative organisms. Unfortunately, extensive use of antibiotics is the cause of resistance phenomena, and treatment of various infections faces a serious problem (9, 17).

In this study, the prevalence of ESBL-positive *E. coli* isolates was 25.92%, which is lower than the ESBL production in clinical isolates of *E. coli* reported in Kerman (68%) (17). Najar Peerayeh et al. in Tehran reported that 70% of clinical *E. coli* isolates produced by ESBL. The prevalence of ESBL resistance in European countries of *E. coli *isolates is reported to be around 3.9% with variations between countries (19). Overall, the percentage of fecal ESBL-producing *E. coli* was lower than those found in Thailand (71.25%) (20), China (50.5%) (21) and Egypt (60.9%) (22). However, the percentage of fecal ESBL-producing *E. coli* in developed countries including USA, Italy, and France were reported to be 2.8% and 1.59%, 1.4%, respectively (23, 24). In developing countries, most patients received antibiotics treatment without prescription; such common practices in nearly all developing countries exert a selective pressure on *E. coli,* whereas in more developed countries effective strategies for the control of antimicrobials are present, which effectively prevents the emergence of ESBLs (4).

Several worldwide reports have shown the emergence of *bla*_CTX-M_ producing *E. coli *as a significant pathogen since the turn of the 21^st^ century (25). The results of the present study show that dissemination of *bla*_CTX-M-15_ gene is the leading cause of resistance to β-lactam antibiotics in *E. coli *strains isolated of diarrhea in the Kerman (southeast of Iran). This result was consistent with those of Castanheira et al, who reported CTX-M-type ESBLs in a significant portion (83%) of ESBL-producing *E. coli *isolates (26). Kalantar et al, in Kerman found that from 94 ESBL positive *E. coli* isolates, 23.4% isolates were positive for *bla*_CTX-M _gene (17). The results of the present study indicated an increased prevalence of *bla*_CTX-M _gene (91.07%) in *E. coli* isolates in Kerman region. Another study in Tehran on non-duplicate clinical isolates of *E. coli* (stool, urine, soft tissue, exudates and blood specimens), revealed that 61.8% of the 144 CTX resistant *E. coli *isolates carried the bla_CTX-M-1_ group alleles. None of the isolates were positive for CTX-M groups 2 and 9 (18). McGettigan et al., also reported that 48% of cephalosporin-resistant *E. coli *isolates produced CTX-M-type ESBLs in Philadelphia (27). In Iran, the frequency of ESBL positive isolates of Enteroaggregative *E. coli *in children was 52.7%. The frequency of *bla*_CTX-M_ in these isolates was 63.1%. The results of this study indicate that one *E. coli *isolate carries *bla*_CTX-M-15_ and *bla*_OXA-1_ genes, which is similar to the results of the Peirano et al. (25). 

PER type β-lactamases have a high prevalence in Turkey and it’s possible that the dissemination of them in Western Europe and the Far East is mostly related to the widespread immigration of Turkish nationals (29). Similar to previous studies, we couldn’t find any *bla*_PER_ among the *E. coli *isolates in Iran and Thailand (29, 30).

Metallo-β-lactamases have been reported from many countries, particularly in Gram-negative organisms like *P. aeruginosa *and Acinetobacter species. None of MBL producing *E. coli* was detected in this study. In their study, Khoshvaght et al. has evaluated the presence of *bla*_IMP_, *bla*_VIM_ and *bla*_NDM-1_ genes from EAEC isolates and founded that none of the isolates possessed these genes (29).

Phylogenetic analyses in the current study showed that *E. coli *isolates fall into four main phylogenetic groups (A, B1, B2, and D), which most diarrhea isolates belong to A and D groups.

In a study from southeast of Iran, diarrheic *E. coli *isolates mostly fell into phylogenetic groups A and D (31). Several studies indicated that diarrheagenic *E. coli *strains belong to groups A, B1 and D (10, 15). Tropical populations strains associated with severe diarrhea seem to preferentially harbor strains of phylogenetic group A and to a lesser extent B1, these strains might have the necessary genetic background for the emergence of pathogenic intestinal strains. This might be one of the agents explaining the higher prevalence of diarrhea in tropical areas (32).

One of the purposes of this study was to determine to ESBLs and MBLs genes in relation to phylogenetic background in *E. coli* isolates from diarrheal patients in southeast of Iran. Recently, Hemati et al, in northwest of Iran reported that diarrheic *E. coli* isolates were positive for ESBL genes (*bla*_TEM_ and *bla*_SHV_) which, mostly belonged to phylo-groups D and A (33). To the best of our knowledge, there is not any study reported in Iran about ESBL genes such as *bla*_CTX-M-15_ and *bla*_OXA-1 _genes in relation to phylogenetic groups. On the other hand, there are several reports on the presence and prevalence of ESBL genes in relation to phylogenetic background in different geographical areas around the world. In Latin America, Pallecchi et al, found that most of the CTX-M-producing isolates belonged to phylogenetic groups A, B1, D and B2, respectively (34). Blanco et al., reported a high frequency of *bla*_CTX-M__-__14_, *bla*_CTX__-__M-15_ and *bla*_CTX-M-32_ genes in relation to B2 phylogenetic group in *E. coli* isolates in Spain (35). A study on ESBL-producing *E. coli *isolates recovered from blood between 2000 to 2010 of Canadian cases indicated that the majority of isolates belonged to phylogenetic groups (B2, D, A and B1) (25). The presence of *bla*_CTX-M-15_ gene in all major phylogenetic groups reflects the widespread of these enzymes across the diversity of *E*. *coli *strains. However, findings of the current study indicated that most group B2 and B1 phylotypes were devoid of *bla*_CTX-M-15_ gene. The hypothesis might explain this observation, which group B2 might be less exposed to antibiotics than groups A, B1, and D. The phylogenetic group B2 strains mostly carry more virulence genes than other phylo-types and mostly belonged to extra-intestinal pathogenic *E. coli*, whereas other phylo-groups are predominant in the fecal *E*. *coli *population of healthy subjects (10). Clermont et al, reported that CTX-M-15-producing *E. coli* is associated with a high level of antibiotic resistance and virulence genes that belonged to B2 phylo-group, showing that under certain selective pressures, the previously observed trade-off between resistance and virulence genes may not apply (36).

In the current study, imipenem was found to be the most active agent, while resistance to cefotaxime was high. Although resistance to cefotaxime is reported to vary in different areas, and imipenem resistance is still very rare around the world including Iran (17).

 According to the broth micro-dilution results, two isolates were resistant to imipenem; however, results of modified Hodge method showed that none of the isolates produce carbapenemase enzyme The modified Hodge test was weakly positive for carbapenemase production by the *E*. *coli *isolate and strongly positive for carbapenemase production by the *Klebsiella pneumoniae *isolate (37).

The results of this study indicate that some ESBL coding genes are widespread among diarrheic isolates. The positive isolates for *bla*_CTX-M-15_ gene are usually distributed among phylogenetic groups other than B2 and B1 phylo-groups, whereas ESBL-producing *E*. *coli *isolates were disseminated among various phylogenetic groups. Further studies and detailed analysis of the data are necessary to detect the ESBL genes in relation to phylogenetic background.
